# Predictors of mortality among children on Antiretroviral Therapy at a referral hospital, Northwest Ethiopia: A retrospective follow up study

**DOI:** 10.1186/1471-2431-12-161

**Published:** 2012-10-08

**Authors:** Digsu Negese Koye, Tadesse Awoke Ayele, Berihun Megabiaw Zeleke

**Affiliations:** 1 Department of Epidemiology and Biostatistics, College of Medicine and Health Sciences, University of Gondar, Gondar, Ethiopia

**Keywords:** HIV/AIDS, Children, ART, Mortality Predictors, Ethiopia

## Abstract

**Background:**

An estimated 2.5 million children were living with HIV/AIDS at the end of 2009, 2.3 million (92%) in sub-Saharan Africa. Without treatment, a third of children with HIV will die of AIDS before their first birthday, half dying before two years of age. Hence, this study aimed to assess magnitude and predictors of mortality among children on Antiretroviral Therapy (ART) at a referral hospital in North-West Ethiopia.

**Methods:**

Institution based retrospective follow up study was carried out among HIV-positive children from January 1^st^, 2006 - March 31^st^, 2011. Information on relevant variables was collected from patients’ charts and registries. Life table was used to estimate the cumulative survival of children. Log rank tests were employed to compare survival between the different categories of the explanatory variables. Multivariate Cox proportional hazards model was fitted to identify predictors of mortality.

**Results:**

A total of 549 records were included in the analysis. The mean age at initiation of treatment was 6.35 ±3.78 SD years. The median follow up period was 22 months. At the end of the follow up, 41(7.5%) were dead and 384(69.9%) were alive. Mortality was 4.0 deaths per 100 child-years of follow-up period. The cumulative probabilities of survival at 3, 6, 12, 24, and 60 months of ART were 0.96, 0.94, 0.93, 0.92 and 0.83 respectively. Majority (90.2%) of the deaths occurred within the first year of treatment. Absence of cotrimoxazole preventive therapy (adjusted hazard ratio [AHR] = 4.74, 95% CI: 2.17, 10.34), anaemia (haemoglobin level < 10gm/dl) (AHR=2.44, 95% CI: 1.26, 4.73), absolute CD4 cell count below the threshold for severe immunodeficiency (AHR=2.24, 95% CI: 1.07, 4.69) and delayed or regressing developmental milestones at baseline (AHR=6.31, 95% CI: 2.52, 15.83) were predictors of mortality.

**Conclusions:**

There was a high rate of early mortality. Hence, starting ART very early reduces disease progression and early mortality; close follow up of all children of HIV-positive mothers is recommended to make the diagnosis and start treatment at an earlier time before they develop severe immunodeficiency.

## Background

Globally, the HIV pandemic created an enormous challenge to the survival of mankind. By the end of 2008, of the 33.4 million people living with HIV/AIDS worldwide, 15.7 million were women and 2.1 million were children under 15 years of age [1]. An estimated 2.5 million children were living with HIV at the end of 2009 with 92% in sub-Saharan Africa. The number of children receiving ART increased but still more than 2 million HIV-positive children were in need of treatment by 2010 [2]. The number of children under 15 years of age receiving ART increased by 29% between 2008 and 2009. Children represent 6.8% of people receiving ART and 8.7% of those in need [3].

HIV-infected infants and children now survive to adolescence and adulthood. The challenges of providing HIV care are evolving into the challenges of providing both acute and chronic, lifelong care. Recent extensive investments in expanding HIV/AIDS treatment in low- and middle-income countries had resulted a marked improvement in ART coverage from 7% in 2003 to 42% in 2008 [4].

HIV develops very rapidly among infants and children. Without treatment, half of children with HIV will die of AIDS before their second birthday and one third before year one. In 2009, there were 260,000 deaths attributed to HIV in under-15 years children, most of which could have been prevented by early diagnosis and effective treatment. By the same year, only 29% of the 1.27 million children in need of ART in low- and middle-income countries were receiving it [5]. Despite the high risk of early mortality in HIV-infected children, the average age at initiation of therapy in resource-limited settings remained high. For example, in a cohort of more than 2,400 HIV-infected children in West Africa, the average age at start of ART was 4.9 years [6]. Data emerging since the publication of the 2006 WHO recommendations for ART in infants and children approves that early initiation of ART is life-saving [7].

Ethiopia launched the fee-based ART in 2003 and free ART in 2005, delivered as part of the comprehensive HIV/AIDS care [8]. Though evaluation of mortality of HIV-positive children on ART is vital for ART program effectiveness, few studies were conducted so far. Therefore, estimating mortality and its predictors among HIV-positive children would provide an input for policy makers to revise the guidelines of pediatric ART initiation. This will also have implication for program planners and decision makers at various stages of the HIV/AIDS care and support program.

## Methods

Institution based retrospective follow up study was carried out among HIV-positive children. The study was conducted at Felege Hiwot Referral Hospital (Bahir Dar, Ethiopia) from January 1^st^ 2006 - March 31^st^ 2011. Bahirdar is the capital city of Amhara National Regional State and it is located 562 kilo meters from Addis Ababa, capital of Ethiopia. Apart from other services, Felege Hiwot Referral Hospital provides chronic HIV care (both pre-ART and ART) services. There were 1092 children ever enrolled into the HIV care and support program and 661 had ever started ART in the hospital till March 2011.

### ART clinic setup and clinical protocol

The ART clinical services are provided by a team comprising of doctors and nurses. At this clinic, HIV-positive children will be screened for opportunistic infections (OIs), evaluated for clinical staging and eligibility for ART by an ART physician/nurse. ART initiation for infants and children was according to the 2007 guidelines for pediatric HIV/AIDS care and treatment in Ethiopia [9] after thorough adherence preparation and counselling. It is determined clinically (WHO stage) and/or immunologically (CD4 count or percentage). Follow-up appointment depends on the child’s condition ranging from every fortnight to monthly basis.

All HIV-positive children on care and support follow up who had started ART at Felege Hiwot referral hospital pediatric ART clinic were included in the study.

Data were entered and cleaned using EPI info version 3.5.1 and analyzed by SPSS version 16.0 statistical package for windows. Life table was used to estimate the cumulative survival of children and log rank tests were employed to compare survival between explanatory variables. Bivariate Cox-proportional hazards model was fitted for all explanatory variables. Those variables with p-value ≤ 0.2 in the bivariate analysis were fitted to the multivariate Cox-proportional hazards model. Backward LR method was used to select variables for the final model. Hazard ratio with its 95% confidence interval and p-values were used to measure strength of association and identify statistical significant result. P-value < 0.05 was considered as statistically significant association.

### Operational definitions

CD4 count below the threshold for severe immunodeficiency was classified according to the child’s age (for infants CD4<1500/mm^3^, 12–35 months <750/mm^3^, 36–59 months <350/mm^3^ and ≥5 years <200/mm^3^) [9].

Adherence to ART was classified into good, fair or poor by the percentage of drug dosage calculated from the total monthly doses of ART drugs. (Good >95%, fair 85-94%, poor <85%).

Developmental milestone, using the WHO window of achievement, for six gross motor milestones was assessed and classified as: delayed, if a child fails to attain milestones for age; regression, if a child loses what has been attained for age, otherwise normal [9].

Underweight was defined as weight for age Z-score < −2 SD for under-five children and BMI for age Z-score < −2 SD for older children.

Anaemia was defined as having haemoglobin level of less than 10 mg/dl.

### Ethical considerations

Ethical clearance was assured from Institutional Review Board of School of Public Health, College of Medicine and Health Sciences, University of Gondar. Permission letter was obtained from the hospital administration. As this was a retrospective study using secondary data, informed consent from individual patients was not obtained. Names and unique ART numbers were not included in the study.

## Results

### Baseline socio-demographic profile of study participants

Of the 574 children’s records reviewed, 549 were included in the final analysis. The mean age was 6.4 ± 3.8SD years and 60.1% were older than 5 years. About half (52.1%) were males and two third (66.5%) were from urban areas (Table 1).

**Table 1:** **Baseline socio-demographic characteristics of Children on ART at Felege Hiwot referral hospital, January 1**^**st**^**, 2006 - March 31**^**st**^**, 2011**

**Variable**	**Frequency**	**Percent (%)**
**Age**		
< 1yr	22	4.0
1-5 yr	197	35.9
5-15 yr	330	60.1
**Sex**		
Male	286	52.1
Female	263	47.9
**Residence**		
Urban	365	66.5
Rural	184	33.5
**Care giver of the child** (n=452)		
Parents	377	83.4
Guardian	19	4.2
In Orphanage centers	10	2.2
Grand parents	36	8.0
Sibling	10	2.2
**Orphan hood** (n = 438)		
Mother and father alive	241	55.0
Maternal orphan	102	23.3
Paternal orphan	38	8.7
Double orphan	57	13.0

### Baseline clinical and immunological profile of study participants

Majority (96.7%) had appropriate developmental milestones at ART initiation. Nearly two third (68.5%) children started ART at an advanced stage of the disease i.e. WHO clinical stage III or IV. About half of them (52.6%) had absolute CD4 count below the threshold for severe immunodeficiency. About half (52.3%) were on cotrimoxazole prophylactic therapy, 82.5% were underweight and 19.8% had anaemia (<10 gm/dl) (Table 2).

**Table 2:** **Baseline clinical and immunological status of Children on ART at Felege Hiwot referral hospital, January 1**^**st**^**, 2006 - March 31**^**st**^**, 2011**

**Variable**		**Frequency**	**Percent (%)**
**ART eligibility criteria**			
WHO clinical stage		9	1.6
Immunological/CD4Count		35	6.4
Both clinical and immunologic		505	92.0
**Type of drug at initiation of therapy**			
4a(d4T-3TC-NVP)		208	37.9
4b(d4T-3TC-EFV)		37	6.7
4c(AZT-3TC-NVP)		258	47.0
4d(AZT-3TC-EFV)		43	7.8
Kaletra based		3	0.6
**Regimen change during follow up**			
Yes		68	12.5
No		475	87.5
**Absolute CD4 count**			
CD4 count below the threshold		289	52.6
CD4 count above the threshold		260	47.4
**Developmental History**			
Appropriate		531	96.7
Delayed		9	1.6
Regressing		9	1.6
**Cotrimoxazole preventive therapy**			
Yes		287	52.3
No		262	47.7
**Nutritional status**			
Underweight		453	82.5
Normal		92	16.8
Overweight		4	0.7
**WHO clinical staging**			
I		45	8.2
II		128	23.3
III		317	57.7
IV		59	10.7
**Haemoglobin(520)**			
<10gm/dl		103	19.8
≥10gm/dl		417	80.2
**3**^**rd**^**month ART drug adherence (497)**			
Good		491	98.8
Fair		3	0.6
Poor		3	0.6
**OIs during follow up**			
Yes		165	30.1
No		384	69.9
**Side effect during follow up(526)**			
Yes		49	8.9
No		477	86.9

For 12.5% of children on ART, first drug regimen was changed. Drug side effects (63.2%) followed by developing tuberculosis while on ART (20.1%) were the main reasons for changing initial regimen. More than half (53%) of the regimens were changed within the first three months and nearly three quarters (71.2%) within six months of ART initiation. A total of 49 (8.9%) children developed drug side effects, mainly (49%) anaemia followed by Nevirapine associated rash. One hundred sixty five (30.1%) children had one or more major OIs. Tuberculosis was the leading OI (40%) followed by pneumonia (21.8%), oral candidiasis (13.3%) and Pneumocystis jirovecii pneumonia (9.1%).

### Mortality after initiation of ART

The median follow up period was 22 months (ranging between 1 and 62 months, IQR=28 months). At the end of follow up, 384 (69.9%) children were alive, 32 (5.8%) were lost from follow up, 92 (16.8%) were transferred out to other facilities and 41(7.5%) were reported dead. Hence, the risk of death was calculated to be 4.0 per 100 child-years of observation. Concerning the time of death, 26 (63.4%), 31 (75.6%) and 37 (90.2%) of the deaths occurred within the first three, six and twelve months of ART initiation respectively. One in ten deaths (9.8%) occurred after one year of ART initiation. Little information was available about the possible cause(s) of death.

**Figure 1:**
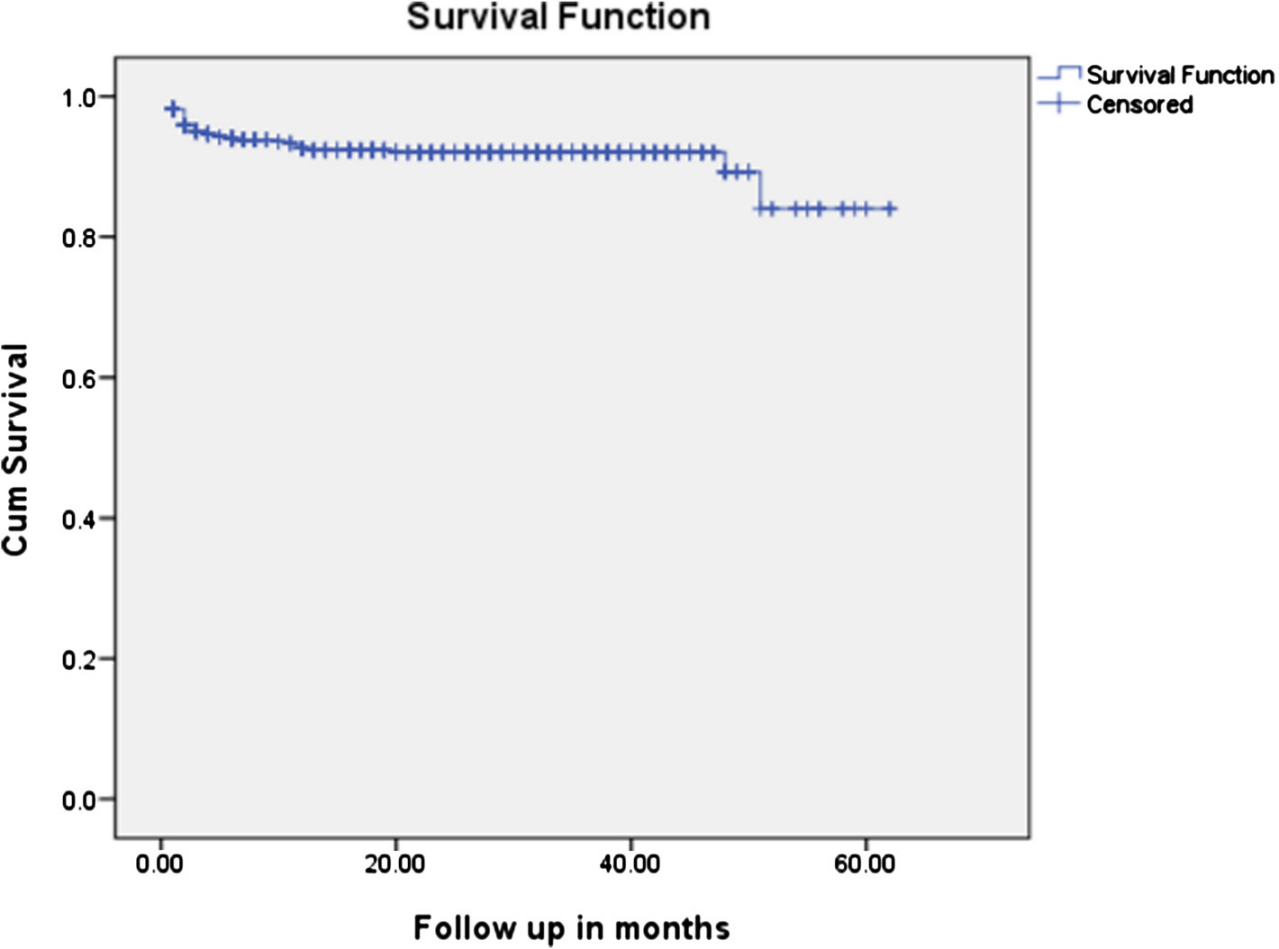
**Kaplan Meier survival curve among Children on ART at Felege Hiwot referral hospital, January 1**^**st**^**, 2006 - March 31**^**st**^**, 2011.**

The mean survival time was 56.5 months (95% CI: 54.62, 58.38 months). For estimation of survival probability two models were used. The first model used confirmed dead cases (n=41) as events (real case assumption) and the second model assumed the worst scenario (worst case assumption) in which both dead and lost cases (n=73) were considered as events. The cumulative probabilities of survival at 3, 6, 12, 24 and 60 months of ART initiation were 0.96, 0.94, 0.93, 0.92 and 0.83 respectively in the real case assumption while it was 0.93, 0.92, 0.90, 0.88 and 0.71 respectively while assuming the worst case scenario (Figure 1).

### Predictors of mortality after initiation of ART

Cox regression revealed that not receiving cotrimoxazole preventive therapy at baseline and delayed or regressing developmental history were predictors of mortality to their counterparts. Similarly, low haemoglobin level and absolute CD4 count below the threshold for severe immunodeficiency were significantly and independently associated with mortality of HIV-positive children. However, age, sex, presence of OI, baseline nutritional status and WHO clinical stage were not significant predictors of mortality.

Accordingly, if the child was not on cotrimoxazole preventive therapy at baseline, there was a 4.74 (95% CI: 2.17, 10.34) times increased risk of death. Children with low haemoglobin level were almost two and half times (AHR = 2.44, 95 CI%: 1.26, 4.73) at risk of death compared to their counterparts. Similarly, children with absolute CD4 count below the threshold for severe immunodeficiency were 2.24 (95% CI: 1.07, 4.69) times at risk of death. A delayed or regressing developmental milestone at initiation of ART 6.31(95 CI%: 2.52, 15.83) times higher risk of death compared to those with appropriate developmental milestone (Table 3).

**Table 3:** **Multivariate Cox regression analysis of predictors of mortality among Children on ART at Felege Hiwot referral hospital, January 1**^**st**^**, 2006 - March 31**^**st**^**, 2011**

**Variable**	**Survival status**		**Crude HR (95% CI)**	**Adjusted HR (95% CI)**
	**Died**	**Censored**		
**Age**				
< 1yr	4	18	1	
1-5 yr	10	187	0.25(0.08, 0.79)	*
5 -15 yr	27	303	0.38(0.13, 1.09)	*
**Sex**				
Male	17	269	0.66 (0.35, 1.22)	*
Female	24	239	1	
**OIs**				
Yes	20	145	2.01 (1.09, 3.72)	*
No	21	363	1	
**Cotrimoxazole preventive therapy**				
Yes	8	279	1	1
No	33	229	4.89(2.26,10.59)	**4.74 (2.17, 10.34)**
**Developmental history**				
Appropriate	34	497	1	1
Delayed/regressing	7	11	10.13(4.43, 23.1)	**6.31(2.52, 15.83)**
**WHO staging**				
I/II	8	165	0.28(0.11, 0.72)	*
III	23	294	0.43(0.21, 0.91)	*
IV	10	49	1	
**Haemoglobin** (n = 520)				
<10gm/dl	15	88	2.87(1.5, 5.5)	**2.44 (1.26, 4.73)**
≥10gm/dl	24	393	1	1
**Nutritional status**				
Underweight	39	414	3.89(0.94, 16.1)	*
Normal	2	94	1	
**CD4 cell count**				
CD4 count below the threshold	30	259	2.55(1.28, 5.1)	**2.24(1.07, 4.69)**
CD4 count above the threshold	11	249	1	1

* Non significant from the multivariate Cox regression (Backward LR method).

## Discussion

At the end of the follow up, there were 7.5% death and 5.8% lost from follow up. The mortality rate was 4/100 child-years of follow up. This was comparable to previous studies in Ethiopia [10,11] and other countries in sub-Saharan Africa [12-16]. In Jimma University hospital [10], 7.3% died and 71.9% were alive at the end of follow up. However, mortality was lower in this study compared to a study from Kenya (8.4%) [17]. This could be explained in three ways. Firstly, the difference in the study period as there were changes in the treatment and care of children on ART through time. Secondly, the Kenyan study was conducted on a lower sample size (n=149) and may be an inadequate estimate. Thirdly, it may be due to the health care system changes in Ethiopia such as decentralization, task shifting and delegation of HIV/AIDS services to low- and mid-level health care providers. Similar to many studies in sub Saharan Africa [10-14,16] where 69% - 89% of deaths occurred in the first six months after ART initiation, in this study more than three quarter of the deaths occurred in the first six months of ART.

This early peak in mortality might be due to delayed ART initiation which increases the risk of severe malnutrition, drug toxicity and immune reconstitution inflammatory syndrome. Moreover, as evidenced from this study, two third of the patients were at WHO clinical stage III or IV and half of them had absolute CD4 count below the threshold for severe immunodeficiency at ART initiation. The study setting was a referral hospital which provides ART and hence children with advanced stages of the disease may be referred in for initiation. The possible reason for increased survival with duration of ART could be the result of progressive increase in CD4 cell count which builds the immune system and the decrease in viral load across time.

As can be noted from the findings of multivariate Cox regression analysis, predictors of mortality were absence of cotrimoxazole preventive therapy, low baseline haemoglobin, absolute CD4 count below the threshold for severe immunodeficiency and delayed or regressing baseline developmental milestone(s). World Health Organization recommends starting cotrimoxazole preventive therapy for all exposed infants at four-to-six weeks of age even before the diagnosis of HIV-infection [18] which is again supported by the result of this study where baseline cotrimoxazole preventive therapy was strongly related to lower risk of mortality. Similarly, data from a large randomized clinical trial in Zambia [19] provided evidence that daily cotrimoxazole preventive therapy is effective in reducing morbidity, mortality, and hospitalizations in HIV-infected children regardless of CD4 value. Cotrimoxazole preventive therapy prevents the development of very serious and fatal OIs [19] and hence it contributes to lower mortality and morbidity.

The other predictor of mortality was CD4 count below the threshold for severe immunodeficiency. This finding was also supported by several studies [7,11-13,20-22].

Lower haemoglobin level (below 10gm/dl) at initiation of ART was another predictor of mortality. Several studies revealed anaemia as an independent predictor of mortality among children on ART [7,11,12,17,21]. Anaemia, a common complication of pediatric HIV infection, is commonly associated with disease progression and death. Late presentation of children for treatment has several associated problems as evidenced from this study (68.4% presented with advanced clinical stage and 82.5% with underweight). Starting ART very early reduces disease progression and early mortality [7].

Having delayed or regressing developmental milestone at initiation of ART was strong predictor of mortality. Delayed or regressing developmental milestone at ART initiation may further complicate the immunological and clinical recovery.

The main limitation of this study is that it includes fewer young children and over-represents children diagnosed at a late stage of the disease. This made comparisons with other similar studies difficult. As data were collected from secondary sources, incompleteness was inevitable and it was difficult to assess clinical and immunological responses. Mortality might be underestimated in this study, since lost to follow-up might also include those died without being reported. The study also failed to assess possible causes of death.

## Conclusions

There was a high rate of early mortality. The mean age at the start of ART was also high. If the child was not on cotrimoxazole preventive therapy at baseline, there exists a higher risk of mortality. Additionally, low haemoglobin level (less than 10gm/dl), absolute CD4 cell count below the threshold for severe immunodeficiency and delayed or regressing developmental milestone at baseline were predictors of mortality.

Hence, starting ART very early reduces disease progression and early mortality; close follow up of all children of HIV-positive mothers is recommended to make the diagnosis and start treatment at an earlier time before they develop severe immunodeficiency. Cotrimoxazole prophylaxis should be initiated as early as possible to all eligible infants and children to reduce early mortality. Children with low haemoglobin level and delayed development shall get proper diagnosis and care such as nutritional interventions to reduce the risk of death.

## Competing interests

The authors declare that they have no competing interests.

## Authors’ contributions

DNK designed the study, performed the statistical analysis and drafted the manuscript. BMZ and TAA participated in the study design, data collection and manuscript writing. All authors contributed to the data analysis, read and approved the final manuscript.

## Pre-publication history

The pre-publication history for this paper can be accessed here:

http://www.biomedcentral.com/1471-2431/12/161/prepub

## References

[B1] UNAIDS Report on the global AIDS epidemic 2009 Geneva, Switzerland: UNAIDS

[B2] Pediatric HIV and treatment of children living with HIVhttp://www.who.int/hiv/topics/paediatric/en/index.html

[B3] WHO, UNAIDS, UNICEF To wards universal access: Scaling up priority HIV/AIDS interventions in the health sector. Progress report 2010 2010 Geneva, Switherland: WHO

[B4] WHO, UNAIDS, UNICEF To wards universal access: Scaling up priority HIV/AIDS interventions in the health sector. Progress report 2009 2009 Geneva: WHO

[B5] Treatment for Children with HIV and AIDS Treatment for Children with HIV and AIDShttp://www.avert.org/hiv-children.htm

[B6] The KIDS-ART-LINC Collaboration Low risk of death, but substantial program attrition, in pediatric HIV treatment cohorts in Sub-Saharan Africa J Acquir Immune Defic Syndr 2008 49 5 523 531 10.1097/QAI.0b013e31818aadce 18989227

[B7] Violari A Cotton MF Gibb DM Babiker AG Steyn J Madhi SA Jean-Philippe P McIntyre JA Early antiretroviral therapy and mortality among HIV-infected infants N Engl J Med 2008 359 21 2233 2244 10.1056/NEJMoa0800971 19020325 PMC2950021

[B8] Department of Disease Prevention and Control Team Guideline for implementation of antiretroviral therapy in Ethiopia January 2007 Addis Ababa, Ethiopia: Ministry of Health

[B9] FMOH-FHAPCO Guidelines for pediatric HIV/AIDS care and treatment in Ethiopia 2008

[B10] Workneh N Girma T Woldie M Immunologic and clinical outcomes of children on HAART: A retrospective cohort analysis at Jimma University Specialized Hospital Ethiop J Health Sci 2009 19 2 75 82

[B11] Taye B Shiferaw S Enquselassie F The impact of malnutrition in survival of HIV infected children after initiation of antiretroviral treatment (ART) Ethiop Med J 2010 48 1 1 10 20607992

[B12] Janssen N Ndirangu J Newell ML Bland RM Successful paediatric HIV treatment in rural primary care in Africa Arch Dis Child 2010 95 6 414 421 10.1136/adc.2009.169367 19880392 PMC3181433

[B13] Bolton-Moore C Mubiana-Mbewe M Cantrell RA Chintu N Stringer EM Chi BH Sinkala M Kankasa C Wilson CM Wilfert CM Clinical Outcomes and CD4 Cell Response in Children Receiving Antiretroviral Therapy at Primary Health Care Facilities in Zambia JAMA 2007 298 16 1888 1899 10.1001/jama.298.16.1888 17954540

[B14] Bong CN Yu JK Chiang HC Huang WL Hsieh TC Schouten EJ Makombe SD Kamoto K Harries AD Risk factors for early mortality in children on adult fixed-dose combination antiretroviral treatment in a central hospital in Malawi AIDS 2007 21 13 1805 1810 10.1097/QAD.0b013e3282c3a9e4 17690580

[B15] Rouet F Fassinou P Inwoley A Anaky M-F Kouakoussui A Rouzioux C Blanche S Msellatif P Long-term survival and immuno-virological response of African HIV-1-infected children to highly active antiretroviral therapy regimens AIDS 2006 20 2315 2319 10.1097/QAD.0b013e328010943b 17117017

[B16] Sauvageot D Schaefer M Olson D Pujades-Rodriguez M O'Brien DP Antiretroviral therapy outcomes in resource-limited settings for HIV-infected children <5 years of age Pediatrics 2010 125 5 e1039 e1047 10.1542/peds.2009-1062 20385636

[B17] Wamalwa DC Obimbo EM Farquhar C Richardson BA Mbori-Ngacha DA Inwani I Benki-Nugent S John-Stewart G Predictors of mortality in HIV-1 infected children on antiretroviral therapy in Kenya: a prospective cohort BMC Pediatr 2010 10 33 10.1186/1471-2431-10-33 20482796 PMC2887829

[B18] WHO, UNICEF Co-trimoxazole prophylaxis for HIV-exposed and HIV-infected infants and children: Practical approaches to implementation and scale up 2009 Geneva, Switzerland: WHO

[B19] Chintu C Bhat GJ Walker AS Mulenga V Sinyinza F Lishimpi K Farrelly L Kaganson N Zumla A Gillespie SH Co-trimoxazole as prophylaxis against opportunistic infections in HIV-infected Zambian children (CHAP): a double-blind randomised placebo-controlled trial Lancet 2004 364 1865 1871 10.1016/S0140-6736(04)17442-4 15555666

[B20] Collins IJ Jourdain G Hansudewechakul R Kanjanavanit S Hongsiriwon S Ngampiyasakul C Sriminiphant S Technakunakorn P Ngo-Giang-Huong N Duong T Long-term survival of HIV-infected children receiving antiretroviral therapy in Thailand: a 5-year observational cohort study Clin Infect Dis 2010 51 12 1449 1457 10.1086/657401 21054181 PMC3106246

[B21] Anaky MF Duvignac J Wemin L Kouakoussui A Karcher S Toure S Seyler C Fassinou P Dabis F N'Dri-Yoman T Scaling up antiretroviral therapy for HIV-infected children in Cote d'Ivoire: determinants of survival and loss to programme Bull World Health Organ 2010 88 7 490 499 10.2471/BLT.09.068015 20616968 PMC2897983

[B22] Musoke PM Mudiope P Barlow-Mosha LN Ajuna P Bagenda D Mubiru MM Tylleskar T Fowler MG Growth, immune and viral responses in HIV infected African children receiving highly active antiretroviral therapy: a prospective cohort study BMC Pediatr 2010 10 56 10.1186/1471-2431-10-56 20691045 PMC2923128

